# Specific gut microbiota and serum metabolite changes in patients with osteoarthritis

**DOI:** 10.3389/fcell.2025.1543510

**Published:** 2025-02-14

**Authors:** Wendong Wang, Xincheng Liu, Hao Nan, Huan Li, Litao Yan

**Affiliations:** ^1^ Department of Engineering Mechanics, Dalian University of Technology, Dalian, China; ^2^ Department of Articular Orthopaedics, The Second People’s Hospital of Dalian, Dalian, China; ^3^ Department of Articular Orthopaedics, The First People’s Hospital of Changzhou, The Third Affiliated Hospital of Soochow University, Changzhou, China

**Keywords:** osteoarthritis, gut microbiota, serum metabolites, multi-omics, correlation analysis

## Abstract

**Introduction:**

Recent research indicated a strong link between the gut microbiota and osteoarthritis. However, the complex interplay between the gut microbiota, serum metabolites, and the progression of osteoarthritis in affected individuals remains largely unexplored. This study aimed to investigate the characteristics of the gut microbiota and serum metabolites in patients with osteoarthritis.

**Methods:**

Participants with either healthy knees or osteoarthritis were enrolled and categorized into healthy control (HC) and osteoarthritis (OA) groups. Fecal and blood samples were collected for 16S rRNA gene sequencing, metabolomic analysis via liquid chromatography–mass spectrometry (LC-MS), and integrated evaluation.

**Results:**

The results showed no significant variation in gut microbiota richness and diversity between the two groups. However, the abundance of *Bacteroides plebeius* and *Faecalibacterium prausnitzii* was reduced in the OA group, both of which are known for their potential as next-generation probiotics for human health. Metabolomic analysis indicated that serum metabolites, including pyrogallol and 3-hydroxybutyrate (3HB), were significantly lower in the OA group. These metabolites are known to positively impact osteoarthritis progression and other diseases and demonstrated good diagnostic performance for distinguishing osteoarthritis patients from healthy controls. Correlation analysis revealed a positive correlation between *Bacteroides plebeius* and *Faecalibacterium prausnitzii* and between pyrogallol and 3HB.

**Discussion:**

This study highlighted specific gut microbiota and serum metabolite profiles in osteoarthritis patients, suggesting that the specific changes in bacteria and derived metabolites are closely tied to osteoarthritis progression. This underscores the potential of gut microbiota and serum metabolites as modifiable elements and therapeutic targets for osteoarthritis prevention.

## Introduction

Osteoarthritis is characterized by synovial inflammation, reactive hyperplasia of articular margins and subchondral bone, and loss and degradation of articular cartilage, making it a degenerative disease prevalent among middle-aged and older populations (Quicke et al., 2022; Sun et al., 2019). Osteoarthritis is a primary contributor to disability and a significant economic burden in the aging population. With an aging and increasingly obese population, this syndrome is becoming more prevalent than in previous decades (Hunter and Bierma-Zeinstra, 2019). Obesity is a key determinant in both the development and worsening of osteoarthritis. Although the main cause of osteoarthritis was traditionally considered to be the overloading of joints due to excess weight, leading to the destruction of articular cartilage, recent studies have shown that other factors such as adipose deposition, insulin resistance, and, particularly, the improper coordination of innate and adaptive immune responses may lead to the initiation and progression of obesity-associated osteoarthritis (Nedunchezhiyan et al., 2022).

The increasing prevalence of osteoarthritis is not only due to longer life expectancy but also due to modern lifestyle factors, including physical inactivity and diets low in fiber and rich in sugar and saturated fats, which promote chronic low-grade inflammation and obesity (Berenbaum, 2019). Adverse alterations in gut microbiota composition, known as microbial dysbiosis, may favor metabolic syndrome and inflammaging, both of which are crucial components in the onset and evolution of osteoarthritis (Biver et al., 2019). The gut microbiome is a complex system that significantly impacts host health, shaped by the interplay of environmental factors and genetic influences. Recently, enteric dysbacteriosis has been identified as a causal factor in the initiation and propagation of obesity-associated osteoarthritis in animal models (Liu et al., 2019). Gut microbes and their components, as well as microbe-associated metabolites, interact with osteoarthritis at both systemic and local levels through mechanisms involving the innate immune system. Metabolites produced by commensal gut microbes influence host health through their recognition by the immune system and their impact on numerous metabolic pathways (Brown et al., 2023). However, the demonstration of causality in humans will require further studies.

To study complex biological processes holistically, it is imperative to adopt an integrative approach that combines multi-omics data to highlight the interrelationships of the involved biomolecules and their functions (Subramanian et al., 2020). The interplay between gut microbiota and serum metabolites offers a more nuanced view of the gut microenvironment’s role in osteoarthritis progression. Elucidating the intricate mechanisms linking gut microbiota, serum metabolites, and osteoarthritis progression is crucial. This interplay could uncover new avenues for investigating the pathogenesis of osteoarthritis and identifying early diagnostic biomarkers, potentially leading to innovative therapeutic strategies to enhance the health status of aging populations. Currently, our understanding is limited by the scarcity of the literature on this topic, indicating a significant gap that warrants further exploration.

Our research aimed to define the gut microbiota and serum metabolite profiles in osteoarthritis patients. By utilizing a multi-omics strategy that involved 16S rRNA gene sequencing and LC-MS metabolomic profiling for fecal and serum samples, we sought to establish connections between distinct gut microbiota and associated metabolites with osteoarthritis progression. Through this investigation, we analyzed the characteristics of gut microbiota, serum metabolites, and their interactions in patients with osteoarthritis, contributing to a novel understanding of the disease’s underlying mechanisms and potential treatment options.

## Materials and methods

### Study subjects and sample collection

**FIGURE 1:**
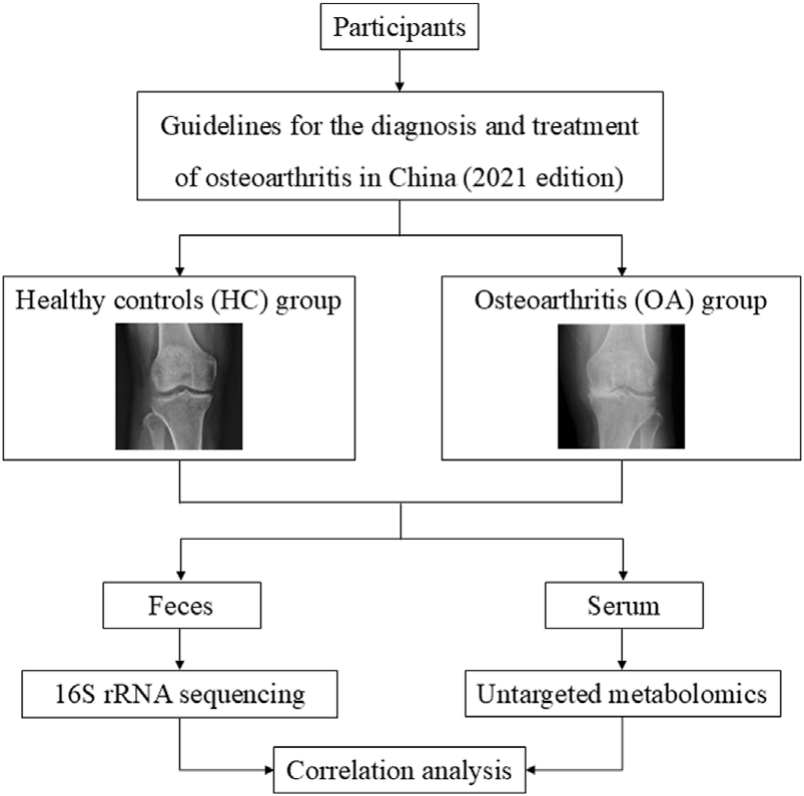
Flow diagram of this study.

A cohort of 38 individuals, comprising 15 healthy controls (HC group) and 23 osteoarthritis patients (OA group), was enrolled at The Second People’s Hospital of Dalian. Basic clinical data, such as age, height, weight, and body mass index (BMI), in the OA group were matched with the HC group, with no significance. Detailed clinical data are provided in Supplementary Table S1. Ethical approval for the study was granted by the Ethics Committee of The Second People’s Hospital of Dalian (No. 2022.174X). All the patients fulfilled the guidelines for the diagnosis and treatment of osteoarthritis in China (2021 edition). Exclusion criteria included individuals with cancer, renal disorders, metabolic or genetic bone conditions, gastrointestinal diseases, mental health issues, or recent antibiotic use within 3 months. Feces and serum samples were obtained from participants and stored at −80°C for subsequent analysis. The study process is delineated in Figure 1.

### Microbiota 16S rRNA gene sequencing

The V4 region of the bacterial 16S rRNA gene was targeted for amplification with primers 515F and 806R. The sequencing libraries were prepared using the TruSeq DNA PCR-Free kit with Illumina index codes. Sequencing was performed on an Illumina NovaSeq platform, yielding 250-bp paired-end reads. The QIIME V.2.0 Pipeline was used after barcodes were taken off the sequences (Hall and Beiko, 2018). A random selection of reads was used to reduce the ASVs in each sample to 10,000 (Valles-Colomer et al., 2022). ASV taxonomy was assigned based on the Silva 138 SSURef NR99 16S rRNA gene reference database (Quast et al., 2013; Zhao et al., 2022).

### Analysis of gut microbiota profile

Alpha diversity of gut microbiota was estimated using R package Vegan (v2.5-7), and the beta diversity was estimated using the vegdist function of the Vegan R package based on the genus levels’ Bray–Curtis distance matrix (Lloyd-Price et al., 2019). The significant difference in beta diversity was assessed using the PERMANOVA test (adonis function), and the bacterial composition was analyzed using the phyloseq R package (v1.50.0) (McMurdie and Holmes, 2013). Furthermore, the Linear discriminant analysis Effect Size (LEfSe) analysis was performed using the microbiomeMarker R package (v1.11.0) (Cao et al., 2022). Moreover, the predicted function of microbiota was estimated by PICRUST2 (Phylogenetic Investigation of Communities by Reconstruction of Unobserved States 2) (v2.5.2), which used machine learning approaches to predict the function of microbiota by matching 16S rRNA sequences with known functional gene sequences (Douglas et al., 2020; Liu et al., 2016).

### Metabolite extraction and LC-MS analyses

LC-MS analyses were conducted on a Vanquish UHPLC system (Thermo Fisher, Germany) interfaced with an Orbitrap Q Exactive™ HF Mass Spectrometer (Thermo Fisher, Germany). The samples were loaded onto a Hypersil GOLD Column (100*2.1 mm, 1.9 μm) with a 12-min gradient elution at a flow rate of 0.2 mL/min. For positive mode (PIM), the mobile phases were A (0.1% formic acid in water) and B (methanol). For negative mode (NIM), the phases were A (5 mM ammonium acetate, pH 9.0) and B (methanol). The gradient program was as follows: 2% B for 1.5 min, increasing to 85% B over 3 min, then to 100% B over 10 min, back to 2% B over 10.1 min, and maintaining 2% B for the final 12 min. The Q Exactive™ HF Mass Spectrometer operated in both positive and negative modes, with a spray voltage of 3.5 kV, a capillary temperature at 320°C, a sheath gas flow at 35 psi, an auxiliary gas flow at 10 L/min, an S-lens RF level at 60, and an auxiliary gas heater temperature at 350°C.

### Data processing and metabolite identification

LC-MS-derived raw data were analyzed using Compound Discoverer 3.3 for metabolite peak alignment, identification, and quantification. Parameter settings included peak area correction against the initial QC sample, a mass accuracy of 5 ppm, a signal-to-noise threshold of 30%, and an intensity floor. Normalized peak intensities were scaled to the overall spectral intensity. This normalization allowed for molecular formula prediction from ion adducts, parent, and fragment ions. Peak matching against mzCloud, mzVault, and MassList databases ensured precise identification and quantification. Statistical processing was executed with R (v3.4.3), Python (v2.7.6), and CentOS (v6.6). Non-normal distributions were adjusted using the following formula: (raw quantitation value/(total sample metabolite values/total QC1 metabolite values)) to determine relative peak areas. Compounds with QC sample CVs over 30% were filtered out, culminating in the definitive identification and quantification of the metabolites.

### Analysis of serum metabolomics

Data including mass-to-charge ratios (m/z), retention times (RT), and normalized peak areas were imported into SIMCA for metabolite identification, which was further aided by the KEGG database and HMDB (Kanehisa et al., 2023; Wishart et al., 2022). The R package ropls (v1.38.0) was utilized to detect intra-group metabolic changes, and partial least squares discriminant analysis (PLS-DA) was employed to assess the prevalence of significant metabolites. Metabolites were deemed differential if they exhibited variable importance in projection (VIP) score >1, p-value <0.05, and fold change >1.2. Volcano plots, generated with ggplot2 in R, were applied to filter metabolites of interest based on log2 (fold change) and -log10 (p-adj). Additionally, pathway enrichment analysis between the HC and OA serum metabolite profiles was conducted using the KEGG database. A linear regression model based on the signatures of metabolites was built (Liang et al., 2020) to discriminate OA participants from HC cases in both the discovery and validation sets using R package pROC (v1.18.5) (Robin et al., 2011).

### Correlation analysis

Spearman’s correlation was applied to assess the associations between predicted functions and alterations in the microbiota and between these changes and metabolites. The analysis was conducted using R package psych (v2.4.1).

### Statistical analysis

All statistical analyses and graphical representations of the study were performed using the R package. Data were expressed as the mean ± SEM unless otherwise stated. A t-test was used to determine significant differences in continuous variables among groups, with a statistical significance assessed at a confidence level of 0.05.

## Results

### Characteristics of the gut microbiota profile

**FIGURE 2:**
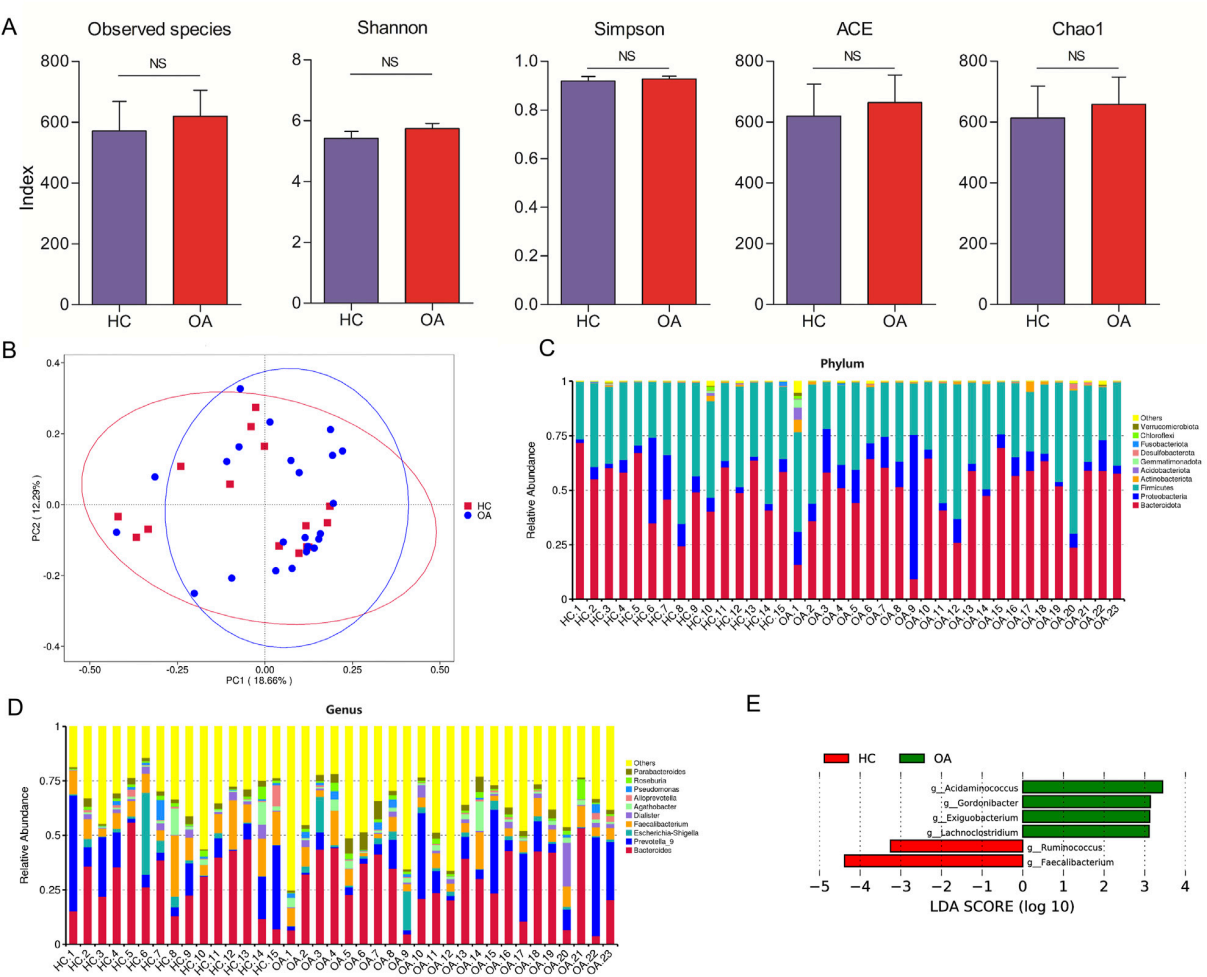
Characteristics of the gut microbiota composition. **(A)** Alpha diversity comparison of microbiota between the two groups. **(B)** PCoA analysis of gut microbiota at the genus level. **(C, D)** Gut microbiota composition in the two groups at phylum and genus levels, respectively. **(E)** LDA scores to identify the different bacteria at the genus level (LDA scores >3).

Figure 2A illustrates the computation of several diversity indices, such as Observed species, Shannon, Simpson, ACE, and Chao1 index, to evaluate the gut microbiota. The findings showed no significant variation in gut microbiota diversity between the two groups. Furthermore, principal coordinate analysis (PCoA) was utilized to examine the distinctions among the sample groups. The PCoA based on genus abundance of the gut microbiota highlighted distinctions between the OA and HC groups, as depicted in Figure 2B. At the phylum level, major bacteria included *Bacteroidota*, *Proteobacteria*, *Firmicutes*, *Actinobacteria*, *Desulfobacterota*, and *Verrucomicrobiota* (Figure 2C). At the genus level, major bacteria included *Bacteroides*, *Prevotella 9*, *Escherichia-Shigella*, *Faecalibacterium*, *Dialister*, *Agathobacter*, *Alloprevotella*, *Pseudomonas*, *Roseburia*, and *Parabacteroides* (Figure 2D).

### Identification of the signatures of the gut microbiota profile and metabolic function

**FIGURE 3:**
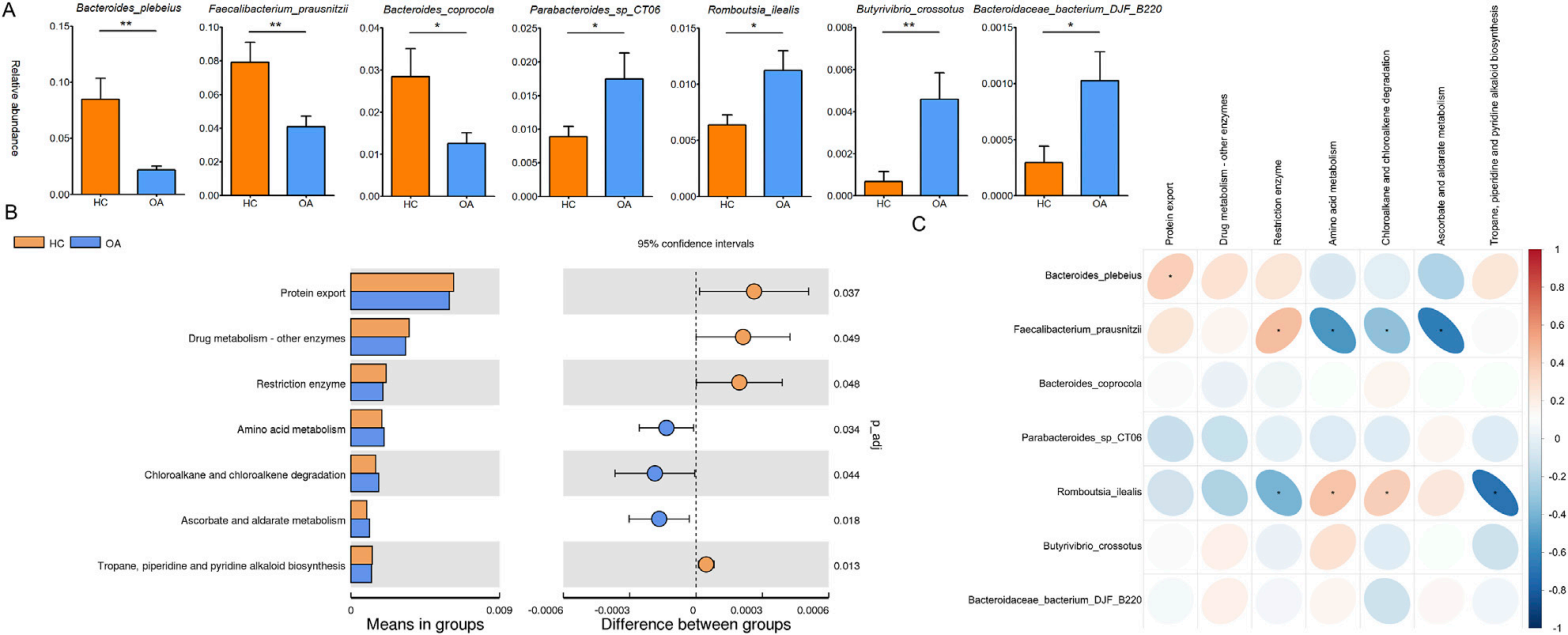
Characteristics of difference and function of the gut microbiota. **(A)** Significant difference in the gut microbiota at the species level. **(B)** Significant difference in bacterial predicted functions between the two groupsassessed by PICRUST2. **(C)** Correlation analysis of the distinct bacterial predicted functions and bacterial abundance. *, p < 0.05; **, p < 0.01.

LEfSe analysis was conducted to identify genus-level bacterial differences between the two groups. As shown in Figure 2E, the OA subjects were characterized by an enrichment of the genera *Acidaminococcus*, *Gordonibacter*, *Exiguobacterium*, and *Lachnoclostridium*. Meanwhile, decrease in the genera *Ruminococcus* and *Faecalibacterium* was observed in the OA group. At the species level, the abundance of *Bacteroides plebeius*, *Faecalibacterium prausnitzii*, and *Bacteroides coprocola* in the OA group was lower than that in the HC group. However, a higher abundance of *Parabacteroides sp. CT06*, *Romboutsia ilealis*, *Butyrivibrio crossotus*, and *Bacteroidaceae bacterium DJF B220* was observed in the OA group (Figure 3A).

Furthermore, the predicted metabolic function analysis of the gut microbiota showed that the protein export, drug metabolism by other enzymes, restriction enzyme, and tropane, piperidine, and pyridine alkaloid biosynthesis pathways were decreased in the OA group. Meanwhile, the amino acid metabolism, chloroalkane and chloroalkene degradation, and ascorbate and aldarate metabolism were increased in the OA group (Figure 3B). Importantly, *Romboutsia ilealis*, which increased in OA individuals, was positively correlated with the metabolic functions of amino acid metabolism, chloroalkane and chloroalkene degradation, and negatively correlated with restriction enzyme and tropane, piperidine, and pyridine alkaloid biosynthesis pathways. Meanwhile, *Bacteroides plebeius* and *Faecalibacterium prausnitzii*, which decreased in OA individuals, showed a positive correlation with the metabolic functions of protein export and restriction enzyme and a negative correlation with amino acid metabolism, chloroalkane and chloroalkene degradation, and ascorbate and aldarate metabolism pathways (Figure 3C).

### The serum metabolome revealed a distinct metabolism in OA subjects

**FIGURE 4:**
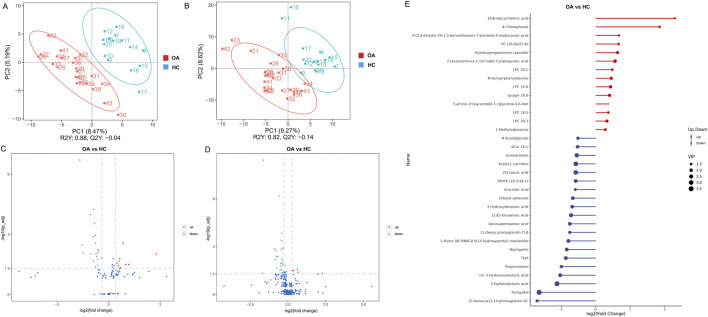
Characteristics of the serum metabolites. **(A, B)** OPLS-DA analysis of serum metabolites between the two groups in NIM and PIM, respectively. **(C, D)** Volcano plots indicated the different metabolites in NIM and PIM, respectively (fold change >1.2). **(E)** Stem diagram indicated the different metabolites between two groups when NIM and PIM are totally combined (VIP >1).

**FIGURE 5:**
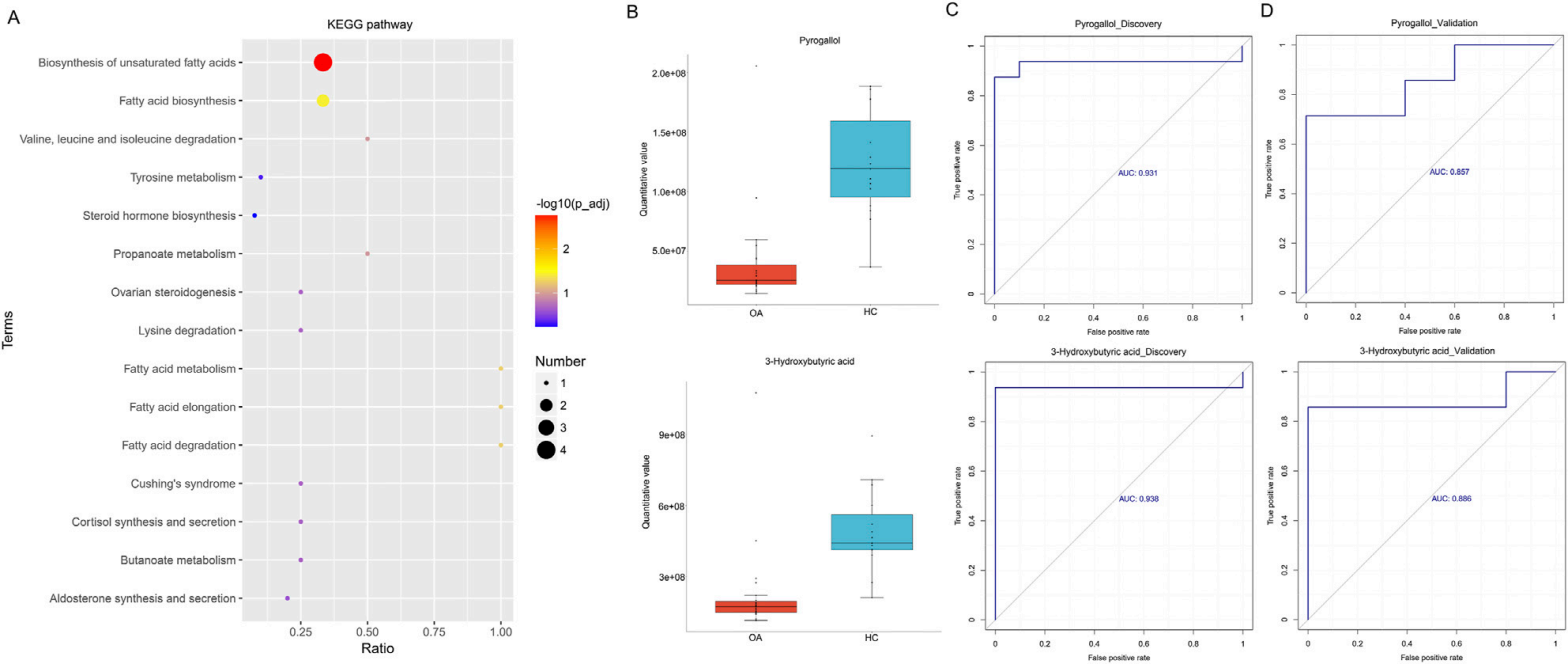
Alterations in serum metabolites associated with osteoarthritis. **(A)** KEGG pathway enrichment based on different metabolites. **(B)** Boxplot indicated the two major differentially enriched metabolites between the two groups. **(C, D)** Linear regression model based on the two major differentially enriched metabolites to discriminate OA from HC subjects in both the discovery and validation sets.

We further analyzed serum metabolites by LC-MS, focusing on NIM and PIM. As shown in Figures 4A–D, both NIM and PIM showed distinct clustering patterns between the OA and HC groups in PLS-DA scores and volcano plots. When NIM and PIM are totally combined, the levels of 14 metabolites exhibited an increase and 20 metabolites exhibited a decrease in the OA group (Figure 4E). Distinct metabolites were significantly enriched in the following metabolite pathways, such as the biosynthesis of unsaturated fatty acids and fatty acids (Figure 5A). Among these distinct metabolites, pyrogallol and 3-hydroxybutyric acid, which were decreased in the OA group (Figure 5B), showed an excellent predictive effect in both the discovery and validation sets (Figures 5C, D).

### Association analysis of the gut microbiota and serum metabolites

**FIGURE 6:**
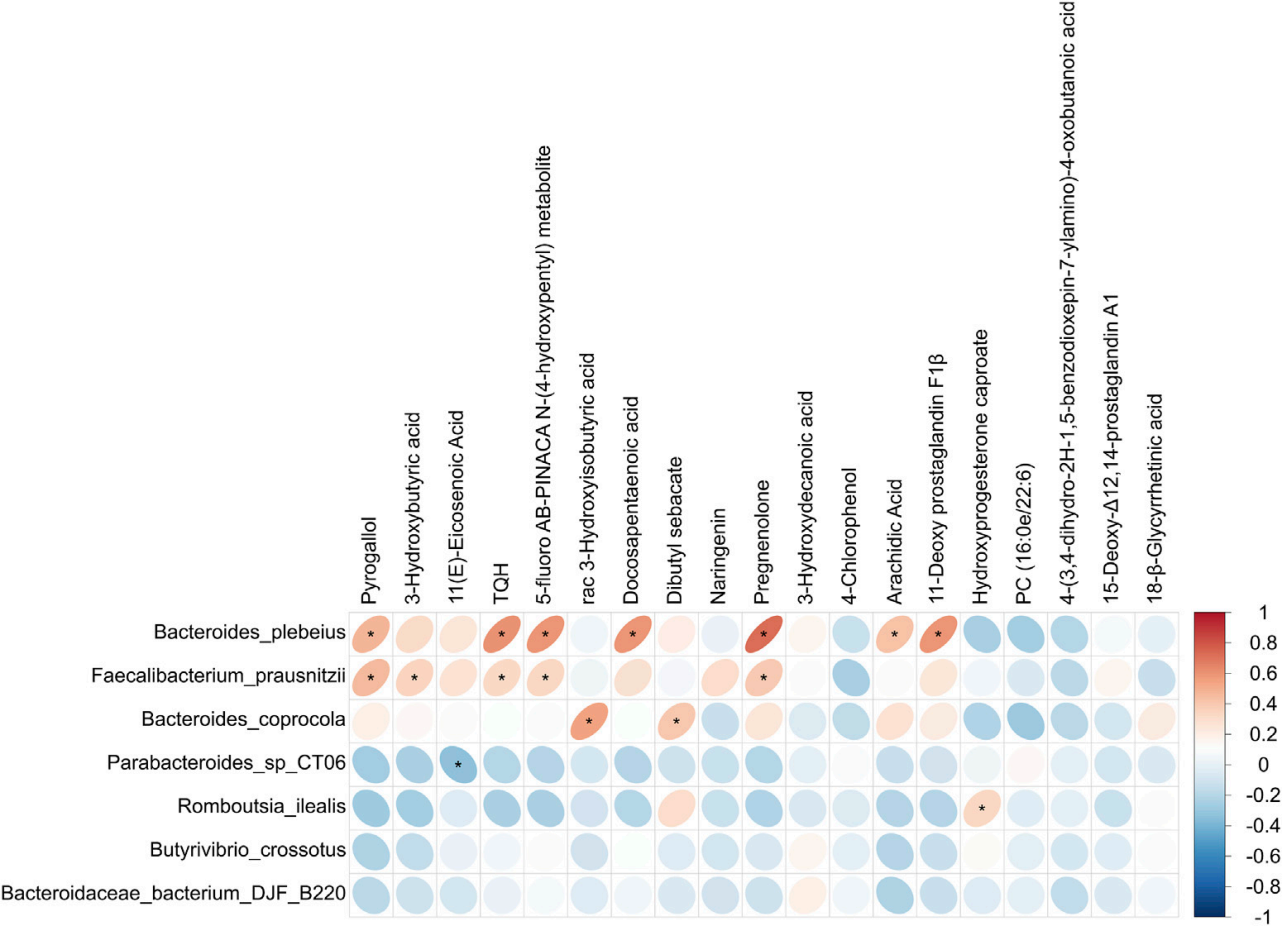
Correlation analysis of distinct gut microbiota and serum metabolites. *, p < 0.05.

A correlation analysis was conducted to explore the link between the differential gut microbiota and serum metabolites, as illustrated in Figure 6. The results showed that *Bacteroides plebeius*, *Faecalibacterium prausnitzii*, and *Bacteroides coprocola,* which were lower in the OA group, were positively correlated with pyrogallol, 3-hydroxybutyric acid, TQH, 5-fluoro AB-PINACA N-(4-hydroxypentyl) metabolite, rac 3-hydroxyisobutyric acid, docosapentaenoic acid, dibutyl sebacate, pregnenolone, arachidic acid, and 11-deoxy prostaglandin F1β, which were also decreased in the OA group. *Parabacteroides sp. CT06*, which was higher in the OA group, showed a negative correlation with 11(E)-eicosenoic acid, which was decreased in the OA group. In addition, *Romboutsia ilealis,* which was higher in the OA group, showed a positive correlation with hydroxyprogesterone caproate, which was increased in the OA group.

## Discussion

Accumulated evidence has shown that dysbiosis of the gut microbiota and metabolites contributes to orthopedic disorders (Yan et al., 2024), but less is known about the gut microbiota and serum metabolite signatures in patients with osteoarthritis. The prevalence of osteoarthritis among the elderly poses a considerable challenge for both patients and healthcare systems. Identifying optimal targets for investigating risk factors and developing novel interventions and treatments for osteoarthritis is crucial. In this study, we analyzed the gut microbiota composition and serum metabolite profiles in osteoarthritis patients. Our findings revealed significant changes in gut microbiota composition and serum metabolite profiles between the OA and HC groups. The correlation between gut microbiota and serum metabolites suggests their potential as modifiable elements and therapeutic targets for the prevention of osteoarthritis.

Alterations in the gut microbiota were associated with osteoarthritis (Chen et al., 2023). The findings of our study suggested that α diversity of the gut microbiota showed no significant change between the OA and HC groups, indicating that bacterial richness and diversity were not significantly altered in the OA group. The results were consistent with those of the previous study (Chen et al., 2023). We focused on the bacterial changes at the species level, such as *Bacteroides plebeius*, *Faecalibacterium prausnitzii*, and *Bacteroides coprocola*, which were enriched in the HC group, while *Parabacteroides sp. CT06*, *Romboutsia ilealis*, *Butyrivibrio crossotus*, and Bacteroidaceae *bacterium DJF B220* were more abundant in the OA group. Notably, the decreased gut bacteria in the OA group were next-generation probiotics with the ability of managing metabolic diseases and producing short-chain fatty acids (SCFAs) (Borgo et al., 2017; De Filippis et al., 2022; Kumari et al., 2021). For example, *Bacteroides plebeius* enabled other *Bacteroides* species to access the sulfated arabinogalactan proteins, providing a route for introducing privileged nutrient utilization into probiotic and commensal organisms that could improve human health (Munoz-Munoz et al., 2021). In addition, *Faecalibacterium prausnitzii* is a typical butyrate-producing bacterium in the gut (Sokol et al., 2008), and butyrate could inhibit the inflammatory response through the NF-κB signaling pathway and promote human health (Segain et al., 2000). *Faecalibacterium prausnitzii* was also reported to be significantly reduced in the gut microbiota of Crohn’s disease patients compared to those in healthy individuals (Takahashi et al., 2016) and attenuates chronic kidney disease (CKD) via butyrate-mediated GPR-43 signaling in the kidney (Li B. et al., 2022). Moreover, *Bacteroides coprocola* and *Faecalibacterium prausnitzii* were regarded as next-generation probiotics, which seem to be very promising as potential preventive and therapeutic agents against obesity and obesity-associated disorders (Vallianou et al., 2023). Proposed mechanisms include the modulation of gut flora and amelioration of intestinal dysbiosis, improvement in intestinal barrier function, reduction in chronic low-grade inflammation, and modulation of gut peptide secretion. However, for the increased gut bacteria in OA subjects, *Romboutsia ilealis* was a potentially harmful bacterium in the gut (Liu C. et al., 2022). In the study about rosmarinic acid alleviating intestinal inflammatory damage by regulating gut microbiota, the abundance of *Romboutsia ilealis* was downregulated by the rosmarinic acid supplement (Li et al., 2023). Consistent with our study, there is a higher presence of *Butyrivibrio crossotus* in the gut microbiota of individuals with periodontal disease than in healthy individuals (Yay et al., 2024). In our research, we conducted a functional prediction analysis of the gut microbiota and discovered pathways that were implicated in the study. In the present study, we also performed a functional prediction analysis of the gut microbiota and found that the pathways involved in the amino acid metabolism, chloroalkane and chloroalkene degradation, and ascorbate and aldarate metabolism were enhanced in the OA group. Conversely, the protein export, drug metabolism by other enzymes, restriction enzyme, and tropane, piperidine, and pyridine alkaloid biosynthesis pathways were decreased in the OA group. Additional correlation studies between gut microbiota and metabolic pathways showed that amino acid metabolism and chloroalkane and chloroalkene degradation were positively associated with *Romboutsia ilealis*, a bacterium that was more abundant in the OA group. Conversely, *Faecalibacterium prausnitzii*, which was less abundant in the OA group, was negatively correlated with these pathways.

The same pattern was observed with the restriction enzyme pathway in relation to *Romboutsia ilealis* and *Faecalibacterium prausnitzii*. These results indicated that some gut bacteria are closely associated with osteoarthritis; however, further studies are needed to determine the underlying mechanisms involved in disease progression.

Furthermore, untargeted serum metabolism analysis was performed, and the results revealed lower levels of 15-deoxy-Δ12,14-prostaglandin A1, pyrogallol, 3-hydroxybutyric acid, rac 3-hydroxyisobutyric acid, pregnenolone, and so on in the OA group than in the HC group. Conversely, higher levels of 18-β-glycyrrhetinic acid, 4-chlorophenol, 4-(3,4-dihydro-2H-1,5-benzodioxepin-7-ylamino)-4-oxobutanoic acid, PC (16:0e/22:6), hydroxyprogesterone caproate, and so on were observed in the OA group than in the HC group. Furthermore, the enrichment of relevant metabolic pathways that differed significantly between the two groups was based on differentially abundant metabolites, which included the enrichment of biosynthesis of unsaturated fatty acids and fatty acid biosynthesis. In terms of decreased metabolites in the OA group, pyrogallol, 3-hydroxybutyric acid, docosapentaenoic acid (DPA), naringenin, and pregnenolone have been reported to be involved in osteoarthritis progression, bone metabolism, or other diseases. Pyrogallol, which was demonstrated to impair staphylococcal biofilm formation via the induction of bacterial oxidative stress (Roese et al., 2023), exhibited antibacterial properties. Calcium silicate-based cement (CSC) has a favorable osteogenic effect in clinical use, and some researchers mixed pyrogallol with bioactive calcium silicate to enhance the antibacterial activity of CSC (Wu et al., 2022). 3-Hydroxybutyric acid, also named β-hydroxybutyrate and 3-hydroxybutyrate (3HB), is a small ketone body molecule produced endogenously by the body in the liver and was demonstrated to reduce the fasting blood glucose level, improve glucose tolerance, and ameliorate insulin resistance in type 2 diabetic mice through hydroxycarboxylic acid receptor 2 (Zhang et al., 2023). Focusing on osteoarthritis treatment, β-hydroxybutyrate has been demonstrated to ameliorate osteoarthritis through the activation of the ERBB3 signaling pathway in mice (Cai et al., 2024). β-Hydroxybutyrate enhances chondrocyte mitophagy and reduces cartilage degeneration in osteoarthritis via the HCAR2/AMPK/PINK1/Parkin pathway (Zhuang et al., 2024), and it alleviates cartilage senescence through the hnRNP A1-mediated upregulation of PTEN (Xia et al., 2023). A recent study reported that gut microbiota-derived 3-hydroxybutyrate blocked GPR43-mediated IL6 signaling to ameliorate radiation proctopathy; the results elucidated the close association between gut microbiota and 3-hydroxybutyrate in disease intervention (Ge et al., 2024). In a study investigating the association between serum polyunsaturated fatty acids (PUFAs) and bone mineral density (BMD), the results demonstrated a linear positive relationship between serum DPA and head BMD, and a non-linear positive association was observed between serum DPA and lumbar spine BMD in US adults (Liang et al., 2023). In addition, DPA was declared to promote human bone-marrow-derived mesenchymal stromal cell (hBMSC) osteogenic differentiation by the miR-9-5p/ERK/ALP signaling pathway, providing a potentially useful therapeutic strategy for patients to improve bone loss (Gao et al., 2022). In a cohort study focusing on the association between the lipid profile and knee and hand osteoarthritis severity, the results showed that the investigated osteoarthritis severity outcomes were associated with the lipidomic fraction of bound and DPA (Loef et al., 2022). For other diseases, the related study indicated that DPA has plasma lipid-lowering and hypoglycemic effects, possibly from the suppression of fatty acid synthesis in the liver (Hirako et al., 2023). These investigations showed the beneficial effects of DPA in orthopedic disorders or other diseases, which was consistent with our study. Naringenin is a citrus flavonoid with anti-inflammatory and antiviral properties, and previous studies have demonstrated its potential capacity in treating lung diseases, including chronic obstructive pulmonary disease, asthma, COVID-19, and lung cancer (Chang et al., 2024). In terms of osteoarthritis, naringenin was reported to attenuate inflammation and apoptosis of osteoarthritic chondrocytes via the TLR4/TRAF6/NF-κB pathway (Wang et al., 2023). In another study, naringenin regulated the production of matrix metalloproteinases in the knee-joint and primary cultured articular chondrocytes, alleviated pain in the rat osteoarthritis model (Wang et al., 2017), and protected against iron overload-induced osteoarthritis by suppressing oxidative stress (Pan et al., 2022). In a study investigating the effect of nano-naringenin on osteoarthritis, nano-naringenin exhibited a potential anti-arthritic activity by reducing the concentrations of serum MMP-3, ADAMTS-5, and joint MDA and increasing the levels of serum TIMP-3 and joint GSH, similar to indomethacin, and the histopathological results confirmed these outcomes. These results showed that nano-naringenin can be considered a natural therapeutic agent for osteoarthritis owing to its antioxidant and anti-inflammatory activities (Shaban et al., 2023). Pregnenolone (Preg) was the grand precursor of most steroid hormones and had been suggested to be a novel anti-osteoporotic agent; the observations showed that Preg inhibited osteoclast differentiation and protected against lipopolysaccharide-induced inflammatory bone destruction and ovariectomy-induced bone loss (Sun et al., 2020).

In terms of metabolic pathways, the biosynthesis of unsaturated fatty acids was one of the most common metabolic pathways dysregulated in patients’ metabolomic profiles with low BMD (Aleidi et al., 2021). In the study focusing on the effect of QingYan formula (QYF) on perimenopausal syndrome rats, QYF administration effectively reduced high bone turnover, repaired trabecular microstructure damage, and regulated the disturbed metabolic pathways including the biosynthesis of unsaturated fatty acids (Zou et al., 2024). Another study revealed that puerarin improved OVX-induced osteoporosis by regulating phospholipid metabolism and biosynthesis of unsaturated fatty acids based on serum metabolomics (Li H. B. et al., 2022). In the study focusing on elucidating the causal relationship between plasma metabolites and the vulnerability to osteoarthritis, metabolic pathway analysis revealed that the pathogenesis of osteoarthritis was specifically associated with fatty acid biosynthesis (Fu et al., 2024), which was consistent with our study. *Bacillus subtilis* and *Enterococcus faecium* treatment attenuated colonic injury and reduced inflammatory and oxidative stress factors in the serum of osteoarthritic rats, and fatty acid biosynthesis was found to be different from those in the control group (Tang et al., 2022). In another study, *Ligusticum wallichii* attenuated IL-1β-induced apoptosis, inflammatory response, and extracellular matrix (ECM) degradation in mouse chondrocytes; metabolic pathways, including fatty acid biosynthesis, were dramatically changed in IL-1β-treated chondrocytes (Wei et al., 2020). Metabolomic analysis of synovial fluids from rheumatoid arthritis patients showed that fatty acid biosynthesis and unsaturated fatty acid biosynthesis were significantly changed in rheumatoid arthritis patients (Wang et al., 2021). These results indicated that the biosynthesis of unsaturated fatty acids and fatty acid biosynthesis were closely associated with osteoarthritis progression.

In our study, correlation analysis was performed to identify the associations between gut bacteria and serum metabolites. Positive associations were found between *Bacteroides plebeius*, *Faecalibacterium prausnitzii*, and pyrogallol and between *Faecalibacterium prausnitzii* and 3HB. Pyrogallol and 3HB have been reported to show beneficial effects in osteoarthritis progression and other diseases, and they showed excellent diagnostic performance for discriminating individuals with OA from HC individuals. *Bacteroides plebeius* and *Faecalibacterium prausnitzii* have been regarded as next-generation probiotics. Moreover, emerging studies have shown that pyrogallol and 3HB were metabolites derived from the gut microbiota (Ge et al., 2024; Liu Y. et al., 2022). To the best of our knowledge, the association between *Bacteroides plebeius* and *Faecalibacterium prausnitzii* and pyrogallol and 3HB has never been investigated in osteoarthritis progression, and the results have caught our attention and prompted further research plans. In future experiments, the exploratory nature of the correlations between specific bacteria and metabolites will be investigated, and proposed mechanistic studies will be conducted. Due to the limited sample size affecting the statistical power and robustness of the results, larger, more diverse cohorts are necessary to enhance the study’s external validity and generalize the findings to broader populations. Among the elderly, the frequent occurrence of osteoarthritis imposes a considerable challenge on patients and healthcare systems. Consequently, accurately forecasting the onset of osteoarthritis in its early stages is of utmost importance.

## Conclusion

Overall, our findings indicated that osteoarthritis patients exhibited substantial alterations in both gut microbiota and serum metabolites, with a notable correlation between them. This relationship offered insights into the mechanisms underlying osteoarthritis progression and suggested potential biomarkers for early diagnosis. Our research could provide a basis for innovative therapeutic approaches to enhance bone health in the elderly.

## Data Availability

The data presented in the study are deposited in the NCBI repository, accession number PRJNA1213153.
