# Supplementary material for: Specific gut microbiota and serum metabolite changes in patients with osteoarthritis
Source: Front Cell Dev Biol. 2025 Feb 14;13:1543510. doi: 10.3389/fcell.2025.1543510 (PMC11868077; doi:10.3389/fcell.2025.1543510)
Supplement: Supplementary file 1 [file Table1.docx]

**Table S1.** Clinical characteristics of subjects in each group

| **Characteristics** | HC group (n=15) | OA group (n=23) | P-value |
| --- | --- | --- | --- |
| **Age (years)** | 62.60±7.917 | 67.30±5.022 | NS |
| **Height (cm)** | 167.93±8.762 | 168.44±7.519 | NS |
| **Weight (kg)** | 65.53±11.507 | 73.52±12.482 | NS |
| **BMI** | 22.34±2.219 | 25.88±4.221 | NS |

Values are mean ± SD for continuous variables.
